# Phenotypic and genotypic (exon 28) characterization of patients diagnosed with von Willebrand disease type 1 in Eastern Saudi Arabia

**DOI:** 10.25122/jml-2022-0276

**Published:** 2023-03

**Authors:** Faisal Mousa Alzahrani, Asma Abdulrazaq Al Faris, Saeed Sattar Shaikh, Fathelrahman Mahdi Hassan, Maryam Ahmed Aldossary, Osama Al Sultan, Nasreldin Elhadi, Sulaiman Salman Alabsi, Mohammed Alsahli, Layla Abdulmohsen Bashawri, Muzaheed Muzaheed, Anne Goodeve

**Affiliations:** 1 Department of Clinical Laboratory Sciences, College of Applied Medical Sciences, Imam Abdulrahman Bin Faisal University, Dammam, Saudi Arabia; 2 Department of Hematology and Immunohematology, College of Medical Laboratory Science, Sudan University of Science and Technology, Khartoum, Sudan; 3 Department of Internal Medicine, King Fahad Hospital of the University, Imam Abdulrahman Bin Faisal University, Khobar, Saudi Arabia; 4 King Abdulaziz Medical City, Jeddah, Saudi Arabia; 5 Department of Medical Laboratories, College of Applied Medical Sciences, Qassim University, Buraydah, Saudi Arabia; 6 Medical School, University of Sheffield, Sheffield, United Kingdom

**Keywords:** Von Willebrand disease type 1, phenotype, genotypes, exon 28, Saudi Arabia

## Abstract

Von Willebrand factor (VWF) is a plasma glycoprotein that plays a key role in hemostasis. Mutations in this protein can result in von Willebrand disease (VWD), the most common form of bleeding disorder in humans. Patients with type 1 VWD have a quantitative plasmatic deficiency of normal structural and functional VWF. Our study aimed to investigate the phenotypic and genotypic characteristics of VWD type 1 patients in eastern Saudi Arabia, focusing on exon 28. We included patients previously diagnosed with WWD type 1 at the King Fahad teaching hospital in Al Khobar and their family members. The correlations between various phenotypic data and genotypic (exon 28) were analyzed using statistical software (SPSS) version 21. While these variants were generally considered benign with minor clinical effects, our analysis did identify two pathogenic variants that could lead to severe VWD symptoms. Specifically, we found these two pathogenic variants in three VWD patients from Saudi Arabia, providing essential insights into pathogenic VWD mutations in this population. Our study, therefore, sheds light on the prevalence of VWF variants in the eastern province of the Kingdom and highlights the need for continued research into the genetic causes of VWD in this region.

## INTRODUCTION

Von Willebrand disease (VWD) is a common bleeding condition estimated to affect 1% of the general population. The prevalence of this disease varies across ethnic groups highlighting the importance of understanding the impact on specific populations [1,2]. VWS is classified into three types, all of which affect von Willebrand factor (VWF), a plasma glycoprotein with a high molecular weight that plays a major role in hemostasis. The VWF protein is encoded by the VWF gene, which is located at chromosome 12 (12p13.3) [3], spanning 178 kilobases (KKB) and consists of 52 exons that widely vary in size (from 40 bases to 1.4 kb) [4]. Type 1 VWD is the most common form of hereditary VWD and is inherited dominantly. Patients with type 1 VWD typically present mild bleeding symptoms. However, many external factors could contribute to the disease severity, including ABO blood group, gender, age, estrogen level, hormonal changes, stress, and infections [5,6].

Diagnosing VWD, especially type 1, is challenging due to the large size of the VWF gene and the high number of benign variants identified. Prior genetic studies of VWD have determined and characterized many variants that affect VWF production through different mechanisms [7-9]. These variants include increased protein retention in the endoplasmic reticulum of the endothelial cells and decreased half-life of the protein in the plasma through increasing clearance, among others. In type 1 VWD, the majority of identified variants (70%) were missense. The remaining highlighted variants were affecting splicing (9%), small deletion (8%), nonsense (5%), or small insertions and duplication mutations (2%) [10,11]. To account for the limited research on the topic, the purpose of this study was to conduct a thorough phenotypic and genotypic characterization of individuals who have previously been diagnosed with type 1 von Willebrand Disease (VWD). The study focused on exon 28, a specific segment of the VWF gene, to examine type 1 VWF in the Saudi Arabian population.

## MATERIAL AND METHODS

Twenty-two index cases and their family members, who were receiving care at the King Fahad Teaching Hospital in Al Khobar, participated in this study. We selected index cases based on their previous diagnosis of type 1 VWD. All participants signed informed consent and answered the International Society on Thrombosis and Hemostasis (ISTH) bleeding score questionnaire https://bleedingscore.certe.nl/ [12]. Blood samples were collected from participants and divided into two tubes: one containing sodium citrate and one containing EDTA. The sodium citrate sample was collected for laboratory tests, including activated partial thromboplastin time (APTT), VWF antigen level (VWF: Ag), factor VIII activity (FVIII: C), and Ristocetin cofactor activity (VWF: RCo), all performed as previously described by J. Eikenboom et al. The EDTA sample was used for platelet count evaluation, ABO blood grouping, and genetic analysis. Platelet counts were analyzed using a Beckman Coulter Unicel DxH 800, while ABO blood grouping was conducted using Anti A and Anti B sera. All tests were performed in the hematology and blood bank departments at King Fahad Teaching Hospital (KFTH) according to standard protocols and quality control measures to ensure accuracy and reproducibility. The remaining EDTA sample was stored at -20°C in the university laboratory for genetic testing.

DNA was extracted using the ReliaPrep™ Blood gDNA Miniprep System Kit from Promega Corporation, USA, following the manufacturer's instructions. The primers used in this study (Table 1) were previously used in another study [7] and were supplied by Invitrogen Company by Thermo Fisher Scientific TM, USA, and reconstituted as specified by the supplier. The PCR reagents were purchased from MOLEQULE-ON^®^ Company, and the thermal cycler machine used was a Maxi TM thermal cycler by ESCO Technologies, USA. After that, the PCR product was separated by agarose gel electrophoresis. The agarose gel powder and the 40x Tris-acetate-EDTA (TAE) buffer were purchased from the MOLEQULE-ON® Company. Furthermore, the DNA ladder and DNA loading dye (6x Bromophenol blue) were used. The DNA band with the expected size was excised and purified by MQ PCR/Gel product purification kit from MOLEQULE-ON^®^ Company, following the manufacturer’s instructions.

**Table 1:** List of internal primers used for sequencing of exon 28.

Exon 28		Primer	Product size
A	Forward	CTTGGATGTGGAATGGTCCA	596 bp
	Reverse	CTTCAGCAAGATCGACCGCC	
B	Forward	CAGCAGGCTACTGGACCTGG	557 bp
	Reverse	CGAGATCGTTAGCTACCTCTG	
C	Forward	CAAGCAGATCCGCCTCATCG	596 bp
	Reverse	GAGATCAAGAGGCTGCCTGG	
D	Forward	AACAGGACCAACACTGGGC	434 bp
	Reverse	CTTGGCAGATGCATGTAGCAG	

Purified PCR products were sent for Sanger sequencing by Applied Biosystem ABI 3730xl Capillary DNA analyzer 96. DNA traces were analyzed by the UCSC genome browser https://genome.ucsc.edu/ using the BLAT tool, VarioBox (Bioinformatics. UA. PT, Portugal), the sequence analysis software (ABI, USA), and Mutation Surveyor V4.0.8 (soft Genetics, UAS). Homo sapiens Von Willebrand Factor (VWF), mRNA NCBI reference sequence: NM_000552.4 and Gene Bank ACCESSION NC_000012 REGION: 5949015.6123196, VERSION NC_000012.12 were used as Gen Bank reference for the sequence analysis of VWF gene.

The comparison of blood groups among different case groups was conducted using the Chi-square test. The bleeding score and frequency of symptoms across all study groups revealed that bleeding symptoms were significantly higher in the Index case group (P=0.003), as determined by the Kruskal Wallis H test. Descriptive statistical analysis was performed using mean values (x) and standard deviation (SD) for continuous variables. Median values were used for data with distribution bias (bleeding score and age). The comparative analysis was performed using the Chi-square test with Yates's correction, and Kruskal-Wallis was applied to compare frequencies. P values <0.05 were considered statistically significant.

## RESULTS

The study included a total of 60 participants, including 22 VWD index cases (IC), 23 affected family members (AFM), and 17 unaffected family members (NAFM). Participant characteristics are summarized in Table 2. There were 11 male and 49 female participants, with a significant difference in gender distribution between the groups (P=0.001).

**Table 2:** Participant characteristics.

		Index cases	Affected family members	Non-affected family members	Controls *	P-value
Number of subjects		22	21	17	100	
Gender N (%)	Males	0	7 (33%)	4 (24%)	72 (72%)	0.001
	Females	22 (100%)	14 (67%)	13 (76%)	28 (28%)	
Age	Mean	32.4±12.55	34.4±20.28	32.6±17.86	35.9±11.96	0.664
	Range	6-54	7-69	6-70	18-65	

IC – Index cases; AFM – Affected family members; NAFM – Non-affected family members.

Table 3 shows that the proportion of cases with the O blood group was higher among the AF and IC groups (95% and 86%, respectively) compared to the NAF and control groups. This difference was statistically significant at a 1% level of significance (p= 0.001). The bleeding score was significantly higher in the index case group (P =0 .001). Table 4 shows that the percentage of bleeding symptoms was significantly higher in the Index case group (P =0.003), as determined by the Kruskal Wallis H test. Comparison of all study groups for each laboratory test showed no significant difference in platelet count and APTT (P=0.593 and p=0.590, respectively). However, Ricof: VWAg ratio and FVIII were significantly lower in the index cases and affected family members compared to a non-affected family member and control groups (P=0.001) (Table 5).

**Table 3:** Blood group distribution across study groups.

Case group		Blood group		P-value
		O group	Other	
IC	N	19	3	0.001
	% within case code	86.4%	13.6%	
AFM	N	20	1	
	% within case code	95.2%	4.8%	
NAFM*	N	10	5	
	% within case code	66.7%	33.3%	
CONTROL	N	54	46	
	% within case code	54.0%	46.0%	

**Table 4:** Frequency of symptoms in the study groups.

Type of symptom	IC		AFM		NAFM	
	No.	%	No.	%	No.	%
Epistaxis	7	31%	7	30%	3	17%
Cutaneous bleeding	14	63%	9	39%	5	29%
Oral cavity bleeding	13	59%	10	43%	2	11%
Bleeding after tooth extraction	5	22%	3	13%	2	11%
Bleeding from minor wounds	9	40%	1	4%	2	11%
Menorrhagia	15	68%	5	21%	6	35%
Post-surgery bleeding	3	13%	0	0	3	17%
Post-partum hemorrhage	5	22%	3	13%	0	0%
Muscle hematomas	6	27%	2	8%	0	0%
Hematuria	3	13%	0	0%	1	6%
gastrointestinal bleeding	3	13%	0	0%	0	0%
Hemarthrosis	0	0%	0	0%	0	0%
Central nervous system bleeding	0	0%	0	0%	0	0%
Other bleedings	5	22%	2	8%	1	6%

**Table 5:** Laboratory results of participants.

Number		Index cases	Affected FM	Non-affected FM	Control*	P-value
		22	21	17	100	
Platelets # (140-450) *10^3^/ul	Median	276	273	307	263	0.593
	25^th^-75^th^ percentile	225.5-338	218.7-319.5	216-330	213-308	
APTT(s) (26-40) s	Median	37.1	37.1	35.7	35.6	0.059
	25^th^-75^th^ percentile	35.3-41	34-39.2	34-36.6	33.6-37.9	
VWAg (50-150) U/dl	Median	53	58.5	90	100	0.001
	25^th^-75^th^ percentile	42.2-56.5	46-66.7	76-96	85-130.2	
RiCof (58-172) U/dl	Median	37	41.5	73	92	0.001
	25^th^-75^th^ percentile	27-41.5	33.5-50	64-83	68-133	
Ricof/VWAg	Median	0.69	0.73	0.8	0.94	0.001
	25^th^-75^th^ percentile	0.6-0.85	0.64-0.81	0.77-0.9	0.75-1.18	
FVIII (70-150) %	Median	82.5	84	120	131	0.001
	25^th^-75^th^ percentile	68.4-94.7	68-110.7	110-139	102.7-169	

In this study, a total of 17 different variants were identified, with 16 variants located in exons and 1 in introns (Table 6). Most of the exonic variants were missense variants that caused a change in the amino acid sequence. There were 11 missenses, and 6 were synonymous variants identified. All the variants identified in this study are known and reported variants. Most of the variants are normal, benign variants that are clinically insignificant, except for 2 variants that were considered pathogenic. Phenotypic and genotypic results showed that all participants had at least 1 variant, but most had more than one variant. All index cases were previously diagnosed with type 1 VWD, while the classification of most family participants was inconclusive, and some had borderline results that made their classification difficult. Only three participants had pathogenic variants that could explain the mutational cause of the disease (Table 7), while the causative disease mutation in most participants was not identified. This may be because the causative disease mutation was associated with other exons in addition to exon 28, which was the focus of the present study.

**Table 6:** List of variants identified in exon 28.

Nucleotide change	Location of the variant	Protein change	Rs number	Variant type	MAF % (1000G)	No. of ICs with this variant	Percentage of variants
c.3692A>C	Exon 28	p. Asn1231Thr	Rs61749368	Missense	0.5%	2	3.8%
c.3735G>A	Exon 28	p. Val1245=	Rs148499318	Synonymous	0.1%	1	1.9%
c.3789G>A	Exon 28	p. Ser1263=	Rs199831474	Synonymous	0.3%	3	5.8%
c.3795G>A	Exon 28	p. Pro1265=	Rs2228319	Synonymous	4.4%	3	5.8%
c.3797C>A*	Exon 28	p. Pro1266Gln	Rs61749370	Missense	0.1%	2	3.8%
c.3797C>T*	Exon 28	p. Pro1266Leu	Rs61749370	Missense	0.1%	1	1.9%
c.3835G>A	Exon 28	p. Val1279Ile	Rs61749376	Missense	0.02%	1	1.9%
c.4130C>T	Exon 28	p. Ala1377Val	Rs141211612	Missense	0.1%	1	1.9%
c.4138A>G	Exon 28	p. Ile1380Val	Rs1106311	Missense	3.1%	3	5.8%
c.4141A>G	Exon 28	p. Thr1381Ala	Rs216311	Missense	22.4%	36 homo 17 hetero	31.4%
c.4304A>G	Exon 28	p. Asn1435Ser	Rs11063987	Missense	3%	3	5.1%
c.4414G>C	Exon 28	p. Asp1472His	Rs1800383	Missense	23.3%	23 homo 25hetero	80%
c.4451G>A	Exon 28	p. Gly1484Glu	Rs267603619	Missense	-	1	1.9%
c.4614G>A	Exon 28	p. Thr1538=	Rs748377348	Synonymous	0%	1	1.9%
c.4665A>C	Exon 28	p. Ala1555=	Rs1800384	Synonymous	17.5%	7	14%
c.4693G>T	Exon 28	p. Val1565Leu	Rs1800385	Missense	17.5	7	14%
IVS5053+17T>C	Intron 28	-	-	Intronic	-	-	-

* – P/LP – pathogenic/likely pathogenic.

**Table 7:** Mutant variants identified in type 1 VWD (Exon 28).

Abnormal mutant variants	Age	Bleeding score	VWF:Ag IU/dL	VWF:RCo IU/dL	FVIII IU/dL	Blood group
IC						
**c.3797C>A**	42	13	36	18	69	O+ve
AFM						
**c.3797C>A**	37	9	32	16	53	O+ve
NAFM						
**c.3797C>T**	50	2	90	73	97	A+ve

## DISCUSSION

In this study, the predominance of female participants among the index cases (ICs) and most affected family members indicates that the symptoms are more apparent in females, likely due to hormonal causes and greater bleeding problems in females. Additionally, the O blood type was the most prevalent in all sample groups, consistent with previous studies showing a connection between blood group and VWF levels. In healthy individuals with the O blood group, the VWF level is 25%, relatively low compared to other blood groups, which can result in lower Ag rates in O-group VWD patients and more serious bleeding symptoms [13]. The bleeding score was significantly higher in the ICs than in other studies (p<0.001), likely due to the lowered blood level of VWF. The incidence of bleeding symptoms was consistently higher in ICs, with menorrhagia, cutaneous bleeding, and epistaxis being the most common symptoms, respectively. In contrast, oral cavity bleeding, cutaneous bleeding, and epistaxis were the most common bleeding symptoms in the affected family members and children, which is consistent with other studies [14]. There were no significant variations in the platelet count groups and the APTT outcomes (p>.005). However, the VWF: Ag, VWF: RCO and FVIII were considerably lower in ICs and AFM, especially when compared to UAFMs and controls (p<.001), due to a reduction in blood VWF level. This finding is consistent with previous population studies [7].

The exon 28 genetic analysis revealed the presence of numerous variants in the Saudi population. Most of these variations are normal variations in the VWF gene and do not affect the levels of VWF in the blood, while others have unknown significance or a pathogenic effect.

Exon 28 analysis revealed the identification of 17 variants, including one intronic variant. Of these, 11 variants (c.3692A>C), (c.3789G>A), (c.3795G>A), (c.4130C>T), (c.4138A>G), (c.4141A>G), (c.4304A>G), (c.4414G>C homozygous), (c.4414G>C heterozygous), (c.4665A>C) and (c.4693G>T) were found to be benign. However, the heterozygous variant (c.3797C>A) (rs61749370) was identified as a likely pathogenic variant in one IC in family 3 in this study. This missense mutation results in a change of amino acid from Proline to Glutamine and has been reported in some patients with type 2M VWD.

Another variant, (c.3797C>T) (rs61749370), was identified in one AFM in family 9 in heterozygous form. This variant results in a change in amino acid from Proline to Leucine [15] and is associated with atypical type 2B VWD. Patients with this variant had normal platelet count and function. The multimeric analysis was normal, and it caused enhanced platelet aggregation. The bleeding score is lower in patients with this variant [16-18]. Additionally, the variant (c.3835G>A) was identified in one AFM in family 9 in this study. This missense variant causes an amino acid change from Valine to Isoleucine and has been reported in many studies in many patients with type 1, 2, and 3 VWD, although its clinical significance is still uncertain [18,19]. While the pathogenic variants found in exon 28 may explain the mutational cause of the disease in some participants, the causative disease mutation in most participants was not identified. This could be because the disease-causative mutation could be found in exons other than exon 28, which suggests the need to extend the present study to cover all the exons in the VWF gene.

## CONCLUSION

This research has provided valuable insights into the prevalence of VWF variants in the eastern province of Saudi Arabia. Two pathogenic variants were identified in three VWD patients in Saudi Arabia, shedding light on some pathogenic mutations associated with VWD. Moreover, given the possibility of misdiagnosis of VWD, our study highlights the need to explore other potential causes of low VWF levels and bleeding in this population. Our results also align with previous studies that have suggested a link between blood group O and variation in VWF plasma levels. Ongoing research can further classify VWF gene alterations associated with VWD in this population. Overall, this study contributes to the growing body of knowledge on VWD in Saudi Arabia and may have implications for improving the diagnosis and treatment of the disease in the region.

## Data Availability

The data used to support the findings of this study are included in the article.
